# Effect of Surface Functionalization on the Magnetization of Fe_3_O_4_ Nanoparticles by Hybrid Density Functional Theory Calculations

**DOI:** 10.1021/acs.jpclett.2c02186

**Published:** 2022-10-03

**Authors:** Enrico Bianchetti, Cristiana Di Valentin

**Affiliations:** † Dipartimento di Scienza dei Materiali, Università di Milano Bicocca, Via Cozzi 55, 20125Milano, Italy; ‡ BioNanoMedicine Center NANOMIB, Università di Milano Bicocca, 20900Monza, Italy

## Abstract

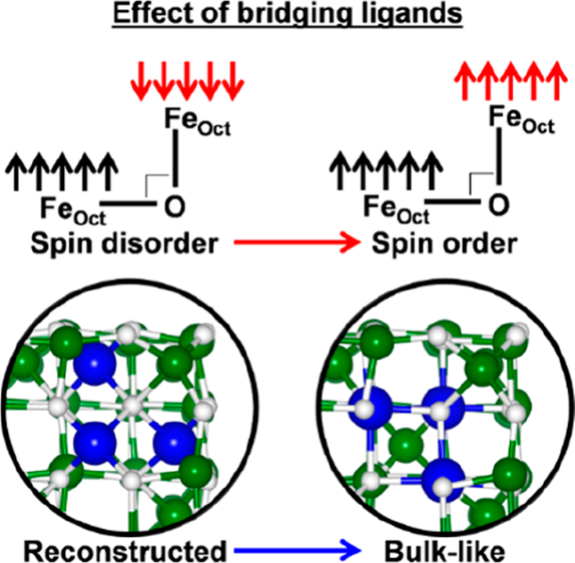

Surface functionalization is found to prevent the reduction of saturation magnetization in magnetite nanoparticles, but the underlying mechanism is still to be clarified. Through a wide set of hybrid density functional theory (HSE06) calculations on Fe_3_O_4_ nanocubes, we explore the effects of the adsorption of various ligands (containing hydroxyl, carboxylic, phosphonic, catechol, and silanetriol groups), commonly used to anchor surfactants during synthesis or other species during chemical reactions, onto the spin and structural disorder, which contributes to the lowering of the nanoparticle magnetization. The spin-canting is simulated through a spin-flip process at octahedral Fe ions and correlated with the energy separation between O^2–^ 2p and Fe_Oct_^3+^ 3d states. Only multidentate bridging ligands hamper the spin-canting process by establishing additional electronic channels between octahedral Fe ions for an enhanced ferromagnetic superexchange interaction. The presence of anchoring organic acids also interferes with structural disorder, by disfavoring surface reconstruction.

Magnetite is the mineral with the highest iron content and with the most intense magnetic properties existing in nature. The inverse spinel crystal structure of Fe(II)Fe(III)_2_O_4_ presents O anions in a face-centered cubic arrangement with the Fe(II) ions in octahedral sites, and the Fe(III) ions half in octahedral and half in tetrahedral sites. The net ferrimagnetic ordering (4.1 μ_B_ per unit formula)^1^ results from the superexchange interaction between the Fe cations through the O anions, which is antiferromagnetic (AFM) for Fe_oct_–O–Fe_tet_ whereas it is ferromagnetic (FM) for Fe_oct_–O–Fe_oct_.^2,3^ The saturation magnetization of bulk magnetite is 96 emu/g.

Magnetite nanoparticles (NPs) can be prepared with different sizes and shapes through controlled synthetic routes^4−9^ and are used in various medical applications as contrast agents for magnetic resonance imaging, in magnetic hyperthermia, or as drug carriers,^10−14^ after being clinically tested and approved for commercialization.

However, the magnetic properties of NPs are not as excellent as those of bulk magnetite, with a saturation magnetization that is largely reduced due to a combination of several contributing factors. The three, generally recognized, main causes of reduced magnetization are the presence of antiphase domains,^15,16^ a low degree of crystallinity, and surface spin-canting effects.^17,18^

Although NPs larger than 80 nm size are usually multidomain,^19,20^ there is still controversy on the minimum size of NPs where multidomains can coexist, depending on the synthetic route or preparation conditions.^16^ In the present work, we will not address this aspect for two reasons: first, because there is reasonable expectation that very small NPs (<25 nm), such as those that are typically used in nanomedical applications, are homogeneously magnetized and are stable single domain;^21^ second, because it has been already theoretically investigated in terms of Heisenberg spin Hamiltonian or through density functional theory with the Hubbard U correction (DFT+U) calculations.^16,22^

A loss in crystallinity at the surface layers has been often invoked as a cause of reduced saturation magnetization in NPs. It is well-known that even extended Fe_3_O_4_ (001) surfaces, which constitute the most stable type of magnetite surface,^23−25^ spontaneously reconstruct through an atomic reorganization where a loss in Fe:O stoichiometry is accompanied by the transfer of some subsurface octahedral Fe ions from the third layer into tetrahedral sites in the second layer.^26^ Indeed, we have shown in a previous study by some of us that a similar reconstruction takes place at the corners of cubic magnetite NPs.^27^ This NP shape is one of the most commonly observed^28^ and is enclosed by six (001) facets.^5,7^ Here, similarly, octahedral Fe ions around the corner move into tetrahedral sites causing structural disorder. However, these surface reconstructions could be hampered using capping agents during NP crystal growth, such as oleic acid, which is often used during NP preparation. However, no theoretical simulations have yet effectively proven this ability of coating polymers to enhance the surface crystallinity of NPs.

Coated magnetic NPs were indeed found to present much higher saturation magnetization, almost close to the bulk value, than naked ones, e.g., 84 vs 46 emu/g.^29−31^ A possible explanation, besides the reduced reconstruction, is that surface coating interferes with spin-canting phenomena, but why and how it could improve the alignment of surface atom spins with the overall magnetization direction of the NP is still a big open question, especially because the covering organic acids are not magnetic. In a recent work,^32^ based on hybrid density functional theory (DFT) calculations on a model of a cubic NP, we have shown that the high saturation magnetization observed for magnetic NPs coated with carboxylic acids is not just simply due to a higher crystallinity of the sample or a reduced disorder at the surface, but it is the consequence of a direct involvement of the carboxylic group in the mechanism of spin alignments. This anchoring functional group bridges pairs of Fe surface ions and becomes involved in a ferromagnetic superexchange interaction between them, which is the reason for the enhanced magnetization with respect to naked NPs but in analogy with the bulk magnetite situation. To corroborate this analysis, based on delicate spin-flipping calculations, we compared the results with those for a nonbridging but still O containing anchoring group, i.e., the hydroxyl group.^33^ Indeed, no ferromagnetic superexchange effect was observed in this case.

Since magnetic NPs are not just coated with hydrophobic organic acids, but eventually those are substituted after synthesis through a “grafting to” approach with many other interesting ligands for a stronger or more efficient surface functionalization,^14^ here we are going to investigate whether the effect that we have observed for the bridging carboxylate group is unique or could be triggered also by other Fe-bridging anchoring groups. If our hypothesis proves true, the protecting polymeric shell not only would provide Fe_3_O_4_ NPs with enhanced dispersion and long-term stability in biological media, together with the possibility of attaching therapeutic agents or targeting agents,^13,14,33^ but also could be used well to improve the magnetic properties by proper tailoring of the grafting strategy.

In the present study we use the protocol developed in our previous work,^32^ based on hybrid DFT calculations, and we apply it comparatively to several different and commonly used anchoring ligands: carboxylic group, phosphonic group, catechol, and silanetriol.^14,33^ At the end of this study, we will also address the role played by the ligands on the NP surface reconstruction.

The hybrid DFT method used is HSE06^34^ because it was proven to reliably reproduce magnetic properties of bulk, surface, and nanostructures of Fe_3_O_4_.^27,35,36^ Further details on the computational setup are provided in the Supporting Information and in refs (27, 35, and 36). The magnetite NP model used in the present study is cubic, is made of 429 atoms, and is enclosed by six (001) facets, as observed in the experiments for the most popular cubic NPs.^5,7^ Such cubic nanostructures were observed to undergo surface reconstruction at four of the eight corners, i.e., those exposing tetrahedral Fe atoms (see Figure 1), from here on referred to as Fe-corner (note that the other four expose O atoms, from here on referred to as O-corner).^27^ Ligand coating for spin-flip calculations is modeled at two different local coverages: low and high. The local low coverage is characterized by one single adsorbed ligand, whereas the local high coverage is by three adsorbed ligands, in the proximity of the Fe ion under investigation. Local high coverage was already proved to well represent the effect of full coverage (when all surface Fe sites are covered by ligands) on spin-flip properties.^32^ Ligand coating for surface reconstruction calculations is modeled at full coverage. The total magnetization is computed according to the formula:^27^
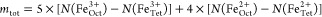
1where Fe_Oct_^3+^ and Fe_Oct_^2+^ are Fe^3+^ and Fe^2+^ ions at octahedral sites, Fe_Tet_^3+^ and Fe_Tet_^2+^ are Fe^3+^ and Fe^2+^ ions at tetrahedral sites, and *N* is the number of the corresponding ions.

**Figure 1:**
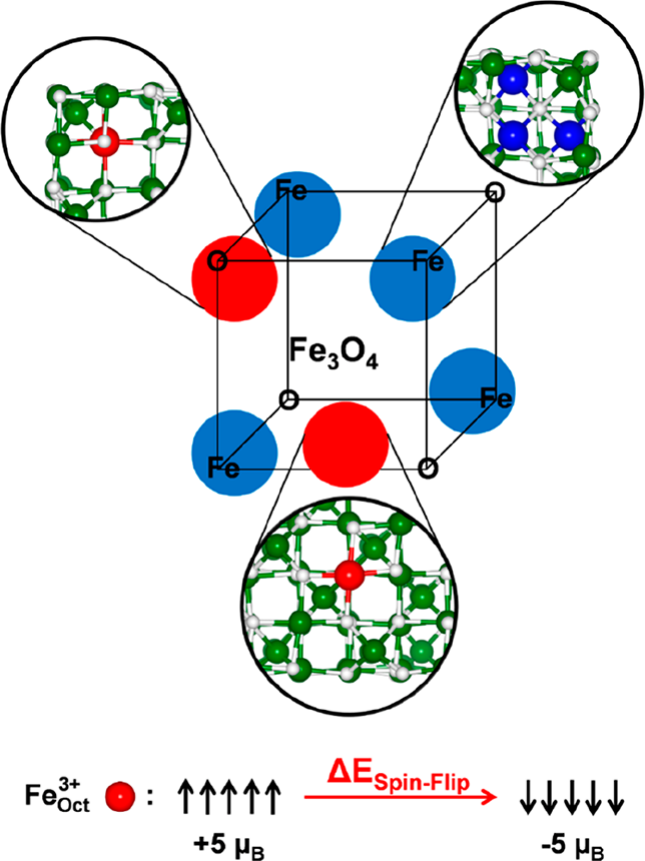
Structural scheme of the magnetite cubic NP. The red circles indicate the corner and the surface portion where the spin-flip process, schematized at the bottom of the figure, is investigated. The blue circles indicate the corners involved in the reconstruction process. The ball-and-stick insets show the details of the surroundings of Fe atoms that are involved in the spin-flip (red beads, which are Fe_Oct_^3+^6*c*-(**3**) in the corner and Fe_Oct_^3+^5*c*-(**5**) on the flat surface, according to the labels used in Figure S1 and in ref (32)) and reconstruction process (blue beads). The white and green beads represent O and Fe.

We mimic the spin disorder, or spin-canting, phenomena by inducing a spin-flip from +5 μ_B_ to −5 μ_B_ on specific Fe_Oct_^3+^ ions^32,37^ (as schematically described on bottom of Figure 1) in the corner and on the flat surface, which are named Fe_Oct_^3+^6*c*-(**3**) and Fe_Oct_^3+^5*c*-(**5**) according to the labels used in Figure S1 and in our previous work,^32^ respectively, as described in Figure 1 and in the corresponding caption. These two specific Fe sites are chosen because the first is a special Fe ion in the proximity of the O-corner, whereas the second is a superficial Fe ion on one of the flat facets. On naked NPs, we have observed in our previous work^32^ that the spin-flip at Fe_Oct_^3+^6*c*-(**3**), followed by atomic relaxation, is characterized by a very tiny energy cost Δ*E*_SF__+Rel_ = Δ*E*_Spin__–Flip_ + Δ*E*_Relaxation_ = +13 meV), and therefore, we expect it can easily take place at room temperature (Table 1). On the flat facet at the Fe_Oct_^3+^5*c*-(**5**) site, the cost is 10 times larger (+137 meV in Table 1) and, therefore, unfeasible at room temperature.^32^

**Table 1:** Selected Nanoparticle Fe_Oct_^3+^ Sites Considered for the Spin-Flip Mechanism Investigation^a^

	coverage	AA	MPA	CAT	SIL	ETH
Fe_Oct_^3+^6*c*-(**3**)	naked	+13^b^ (+270^b^)				
	low	–49^b^ (+80^b^)	–10 (+16)	–27 (+4)	–33 (−1)	–39 (−8)
	high	+141^b^ (+165^b^)	+120 (+130)	+118 (+131)	+134 (+143)	–15^b^ (+15)
Fe_Oct_^3+^5*c*-(**5**)	naked	+137^b^ (+208^b^)				
	high	+280 (+310)	+241 (+275)	+219 (+257)	+209 (+248)	+114 (+210)

a The Δ*E*_SF__+Rel_ and Δ*E*_SF_ (inside the parentheses) values are reported in meV for different ligands at different coverages.

b These values are taken from ref (32).

In contrast, when the NP is decorated with acetic acid (AA) at a high local coverage regime (see Figure 2 and Figure S2), we have observed that the spin-flip process is unfavorable on both Fe_Oct_^3+^6*c*-(**3**) and Fe_Oct_^3+^5*c*-(**5**) and it is accompanied by a small relaxation energy (Δ*E*_SF__+Rel_ = +141^32^ and +280 meV, respectively, see Table 1). As already mentioned in the introduction, the effect of the presence of acetic acid at a high density is related to the ability of this bridging ligand to reconnect several surface octahedral Fe ions and, thus, induce an extra FM superexchange effect. To corroborate this, we also considered a ligand that is not bridging, i.e., ethanol (ETH in Figure 2, dissociating similarly to water molecules^38,39^), and we confirmed that in this case spin-flipping is still an easy process at Fe_Oct_^3+^6*c*-(**3**), as observed for naked NPs (−15 meV vs +13 meV, respectively, as reported in Table 1).^32^

**Figure 2:**
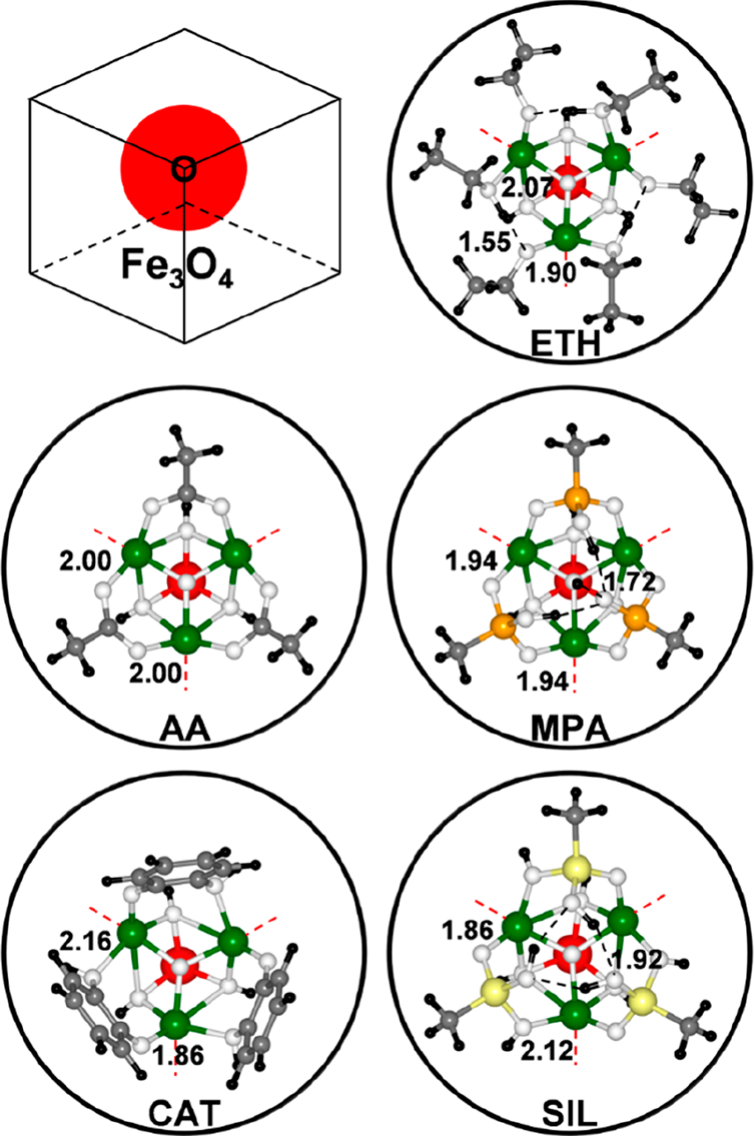
Ball-and-stick representations for the adsorption of different ligands at local high coverage onto the NP corner. Only the ligands and the NP O-corner are shown for clarity, as schematized in the top left corner of the figure. The red dashed lines indicate the edges of the hidden NP. The black dashed lines indicate the formation of hydrogen bonds. The H-bond and the Fe–O_Ligand_ bond lengths (in Å) for the asymmetric unit are shown. The black, gray, orange, yellow, white, green, and red beads represent H, C, P, Si, O, Fe, and Fe_Oct_ on which the spin-flip process is investigated, respectively.

As a further step, we now present the results for other bridging anchoring ligands, starting from phosphonic acid. In particular, we considered methylphosphonic acid (MPA), as the simplest prototypical molecule, in several adsorption modes (see Figure S3 and Figure S4): undissociated monodentate, dissociated chelate, undissociated bidentate, dissociated bidentate, and bidissociated bidentate. The most stable configuration at the O-corner, in the low coverage regime, is the bidissociated bidentate, with an adsorption energy of −5.71 eV (see Figure S3, Figure S4, and Table 2), which is at least 1 eV larger than those computed for the other modes. Note that one of the two dissociated protons goes on the extremely reactive O atom at the O-corner, causing some Fe–O bond breaking and corner reconstruction, whereas the other is adsorbed on an O atom of the flat surface that is generally recognized in the literature as the most easily protonated.^40,41^ If we increase the local density of MPA molecules at the O-corner, in the proximity of the Fe_Oct_^3+^6*c*-(**3**) site (Figure 2), using the three undercoordinated octahedral Fe ions around the O at the vertex, the additional two MPA molecule are found to preferentially adsorb as monodissociated bidentate, which is one of the most common adsorption modes on metal oxide surfaces.^42^ The presence of the other two MPA molecules prevents the Fe–O bond breaking at the O-corner upon OH formation. We also considered MPA adsorption on flat 001 facets, around the Fe_Oct_^3+^5*c*-(**5**) site, with two MPA molecules (Figure S2) in the dissociated bidentate mode (−3.66 eV per molecule).

**Table 2:** Adsorption Energies (in eV per molecule) for Different Ligands at Different Coverages onto the NP

*E*_ads_(eV/molecule)	AA	MPA	CAT	SIL	ETH
corner–low coverage	–2.98^a^	–5.71	–3.91	–4.62	–2.32
corner–high coverage	–3.00	–3.85	–2.66	–3.00	–1.60
surface–high coverage	–2.44	–3.66	–2.55	–2.92	–1.57

a This value is taken from ref (32).

The spin-flipping accompanied by atomic relaxation at Fe_Oct_^3+^6*c*-(**3**) and Fe_Oct_^3+^5*c*-(**5**) sites, when local high coverage of MPA is reached, is clearly much less favorable than for the naked NP, similarly to what observed in the presence of high density of AA: +120 meV and +241 meV, for the two sites, respectively, vs +141 and +280 for AA. Therefore, the bridging ligand MPA [O–P–O], similar to AA [O–C–O], is perfectly capable of activating additional superexchange interaction among octahedral Fe ions.

Next, we consider catechol-based ligands, which are well-known to stably anchor metal oxide surfaces. Again, catechol (CAT) is a bridging ligand [O–C–C–O] that can attach to two surface cations in a bidentate fashion. Indeed, the monodissociated bidentate adsorption mode is found to be that with the largest adsorption energy (−3.91 eV) at the O-corner site (see Table 2, Figure S3, and Figure S5), if compared to undissociated bidentate, bidissociated bidentate, undissociated chelate, dissociated chelate, and bidissociated chelate, which are at least 1 eV higher in energy if not unstable. When three CAT molecules are anchored to the O-corner by using the three octahedral Fe ions around the O at the vertex, we observe a highly symmetric structure, as shown in Figure 2. On the flat 001 facets, catechol molecules are similarly monodissociated and bidentate as on the corner sites (Figure S2).

It is not so easily predictable whether the [O–C–C–O] bridge will be effective in inducing magnetic communication between Fe ions. The results confirm that, in the presence of a high coverage of CAT ligands, spin-flipping, even accompanied by atomic relaxation, is a hampered process by +118 meV at the Fe_Oct_^3+^6*c*-(**3**) site and by 219 meV at the Fe_Oct_^3+^5*c*-(**5**) site. These numbers are slightly smaller than for AA and MPA but are still very close.

The last class of anchoring ligand we have investigated is based on a silanetriol group. In particular, we have considered methylsilanetriol (SIL), which consists of three [O–Si–O] bridges. Similarly to MPA, this ligand could potentially be tridentate; however, it is found to be bidentate because the (001) magnetite surface presents superficial Fe rows that are too distant to allow the tridentation. Here, we observe that at the O-corner SIL is preferentially monodissociated and bidentate on two undercoodinated octahedral Fe ions next to the O at the vertex (see Figure S6), both at low (−4.62 eV) and high (−3.00 eV) coverage densities (see Figure S3 and Figure 2, respectively). We considered other absorption modes, which are at least half an eV higher in energy: undissociated bidentate, bidissociated bidentate, and dissociated chelate.

Are the [O–Si–O] bridges affective for superexchange interaction among octahedral Fe ions? The answer is again positive with an energy cost for spin-flipping, even allowing atomic relaxation, of +134 meV at the Fe_Oct_^3+^6*c*-(**3**) site and by 209 meV at the Fe_Oct_^3+^5*c*-(**5**) site, in line with the other bridging anchoring ligands considered in this study (see Table 1 for direct comparison).

The calculations clearly suggest that all bidentate bridging ligands induce an extra FM superexchange interaction between octahedral Fe ions, which reinforces the overall magnetization of the NPs and suppresses spin disorder phenomena. This effect is evaluated in terms of energy cost (Δ*E*_SF__+Rel_) to induce spin-flip and corresponding atomic relaxation on an octahedral Fe ion in the proximity of the O-corner, where spin and structural reorganization can more easily take place. Larger values of Δ*E*_SF__+Rel_ reflect larger magnetic exchange coupling constants (*J*) between octahedral Fe ions. To understand the origin of this extra FM superexchange, we have analyzed the dependency of *J* with the variation of the O^2–^ 2p band energy levels with respect to the Fe_Oct_^3+^ 3d ones (Δ_pd_), according to the formula:^43^
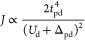
2where *t*_pd_ is the hopping integral between p and d orbitals, and *U*_d_ is the Coulomb repulsion between two electrons in a d orbital, respectively. Similarly to what done in our previous work,^32^ the charge transfer energy Δ_pd_ is estimated as the energy difference between the center of mass (COM) of the O^2–^ 2p-band and of the Fe_Oct_^3+^ 3d-band, as detailed in the SI. Since we simulated a local high coverage of ligands at the O-corner, only the spin-flipping Fe site, Fe_Oct_^3+^6*c*-(**3**), and the O and Fe atoms in its first and second coordination sphere, are considered in the calculation of the O^2–^ p-band and the Fe_Oct_^3+^ d-band COM. We compute a Δ_pd_ of 2.05 eV for the naked NP and of 1.55, 1.49, 1.54, and 1.60 eV for the NP at high coverage of AA, MPA, CAT, and SIL, respectively. According to eq 2, a reduction of Δ_pd_ (here of about 0.5 eV) is expected to enhance the magnetic exchange coupling constant, in perfect agreement with the increase in spin-flip energy cost observed in our calculations, as schematized in Figure 3. This analysis is corroborated also by the opposite case of a local high coverage of ETH, for which we compute a Δ_pd_ value very close to that for the naked NP (1.98 eV vs 2.05 eV, respectively), confirming no additional FM exchange, as suggested by the Δ*E*_SF__+Rel_ value (−15 meV^32^) in favor of spin-flipping.

**Figure 3:**
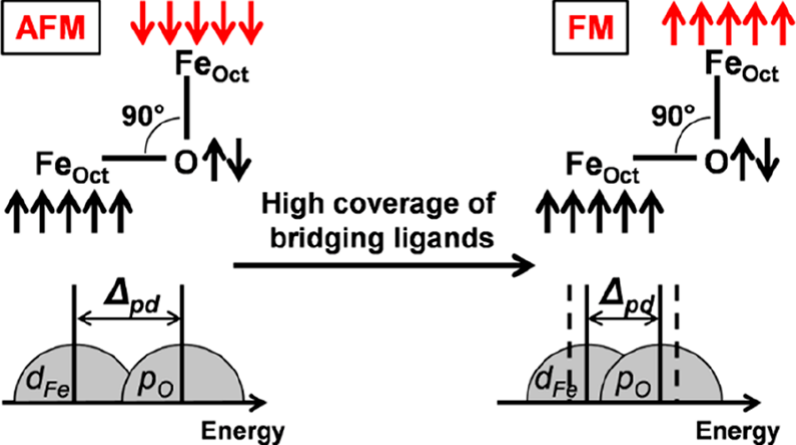
Schematic representation of the extra ferromagnetic (FM) superexchange interaction between octahedral Fe ions induce by adsorbed bridging ligands.

Based on these results and analysis, we can state that an induced extra FM superexchange effect among Fe_oct_ sites in the surface layers is only triggered by adsorption of a bridging ligand that electronically connects different Fe_oct_ sites, such as acetate, phosphonate, catechol, and silanetriol derivates but not monocoordinated molecules, such as ethanol. We further proved this concept by calculating the monodentate AA at local high coverage, as shown in Figure S7. Indeed, we obtain a tiny energy cost of 47 meV for Δ*E*_SF__+Rel_, confirming a cheap spin-flip process, in contrast with what is observed for the bidentate (bridging) mode whose Δ*E*_SF__+Rel_ is three times larger.

In this last paragraph, we wish to get some insight into the role played by ligands on the NP surface reconstruction, since this is one of the major factors affecting the saturation magnetization of NPs, together with spin-canting and the presence of antiphase domain boundaries. In a previous study by some of us,^27^ we have proved that bulk-like cubic NPs are stabilized by surface reconstruction, which consists of the transfer of six-coordinated iron atoms near an Fe-corner from octahedral to tetrahedral sites (see Figure 1). Such an atomic reconstruction was found to reduce the total magnetization of the NP by 9.7% (from 1232 μ_B_ to 1112 μ_B_). In the present study, we have found out that this type of reconstruction stabilizes the cubic NP model under investigation by −10.6 eV. At the top of Figure 4, we show the naked NP before (bulk-like, left) and after (reconstructed, right) the atomic reconstruction, which involves three octahedral iron atoms (blue beads in the image) for each Fe-corner (four out of a total of eight corners per cube). However, if the NP is fully decorated by the organic ligands (AA), we note that the reconstructed model is less stable than the bulk-like one by 7.4 eV (Figure 4, the bottom). We also considered half coverage (see Figure S8), but this is not sufficient to lift the reconstruction. Thus, only high coverage organic acid coating can reverse the energetics with respect to what is observed for the naked NPs, largely stabilizing the bulk-like structure whose total magnetic moment is by far higher than that of the reconstructed NP (408 μ_B_ versus 288 μ_B_, respectively). This finding is in line with what was found by means of surface X-ray diffraction, infrared spectroscopy, and DFT+U calculations by Arndt and collaborators for the (001) surface,^44^ i.e., that the adsorption of organic acid reverses the reconstruction process of the (001) surface, through the stabilization of the bulk-like structure and formation of deep bulk Fe vacancies.^44^ Furthermore, this result suggests that using coating agents during the synthesis of the nanostructures is a successful strategy to prepare NPs with high saturation magnetization, close to the bulk value.

**Figure 4:**
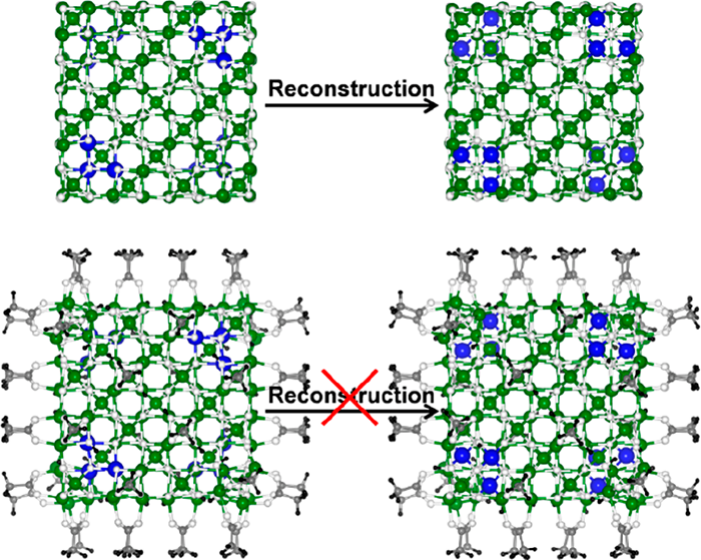
Ball-and-stick representations of the minimum energy structures of the unreconstructed (left) and reconstructed (right) nanoparticle in the absence (top) and in the presence (down) of the acetic acid molecules at full coverage. The black, gray, white, green, and blue beads represent H, C, O, Fe, and Fe_Oct_ which are involved in the reconstruction process, respectively.

In summary, in this Letter we have shed light on the underlying reasons why surface functionalization of NPs keeps saturation magnetization values close to those of the bulk. Not only carboxylic groups, commonly used to anchor surfactants (e.g., oleic acid) during NPs synthesis, but also other kinds of multidentate bridging anchoring groups, which can exchange with surfactants after the synthesis, are found to create electronic channels through chemical bridges between Fe_oct_ sites that induce an extra ferromagnetic superexchange interaction, and work against spin-flipping processes and, consequently, also against the reduction of the total magnetic moments observed for noncoated NPs. Our conclusion is corroborated by the fact that coating with monodentate ligands does not lead to such behavior. In the last part of this Letter, we also show that surface functionalization by organic acids prevents crystallinity loss in NPs, which again improves their total magnetic moment. This is because the surface reconstruction, consisting of octahedral Fe ions that move into tetrahedral sites, is found not to be energetically favorable in the presence of the coating molecules.

To conclude, the results of this computational study provide a first-principles description, at the electronic and atomistic level, of the mechanisms regarding how surface functionalization alters the spin and structural disorder (spin-canting and atomic reconstruction) in magnetite NPs and, consequently, affects their saturation magnetization. Since this specific physical property is crucial for an efficacious nanomedical application, the concepts developed here can be useful to guide the design and preparation of optimal magnetite-based nanostructures.

## Supporting Information Available

Further computational details, global minimum energy structure of the magnetite cubic nanoparticle, figures describing the adsorption of different ligands at local high (low) coverage onto the nanoparticle surface (corner), figures describing the different adsorption modes tested for each ligand, figure of the bulk-like and reconstructed nanoparticles at half acetic acid coverage, and further details regarding the spin-flip of Fe_Oct_ in the naked NP case (PDF)
