# Supplementary material for: Effect of Surface Functionalization on the Magnetization of Fe3O4 Nanoparticles by Hybrid Density Functional Theory Calculations
Source: J Phys Chem Lett. 2022 Oct 3;13(40):9348–54. doi: 10.1021/acs.jpclett.2c02186 (PMC9575150; doi:10.1021/acs.jpclett.2c02186)
Supplement: Supplementary file 1 — jz2c02186_si_001.pdf [file jz2c02186_si_001.pdf]

Supporting Information

Effect of Surface Functionalization on the Magnetization of Fe_3O_4 Nanoparticles by Hybrid Density Functional Theory Calculations

Enrico Bianchetti[a] and Cristiana Di Valentin[a,b1]

[a]Dipartimento di Scienza dei Materiali, Università di Milano Bicocca, Via Cozzi 55, 20125 Milano, Italy

[b]BioNanoMedicine Center NANOMIB, Università di Milano-Bicocca, 20900 Monza, Italy

[1] Corresponding author: cristiana.divalentin@unimib.it

Computational Methods

Hybrid density functional theory calculations (HSE06)[1] were carried out using the CRYSTAL17 package.[2,3] For the validation, against experimental data, of the standard hybrid functional HSE06 as a robust theoretical approach to describe structural, electronic, and magnetic properties of magnetite system, please refer to Ref. 4 and corresponding Supporting Information, where some of us also analyzed the effect of reducing the fraction of the exact exchange, in comparison with B3LYP calculations and PBE+U calculations with different U values. The Kohn–Sham orbitals are expanded in Gaussian-type orbitals: the all-electron basis sets are H|511G(p1), C|6311G(d11), O|8411G(d1) (for NP oxygen atoms), O|8411G(d11) (for adsorbed molecules oxygen atoms), Si|86311(d1), P|8521G(d1), and Fe|86411G(d41). The convergence criterions of 10^{-6} hartree and 4.5×10^{-4} hartree/bohr for total energy and forces, respectively, were used during self-consistent field calculations and geometry optimization, according to the scheme previously used for Fe_3O_4.[4,5,6]

The cubic nanoparticle (NP) model used for this investigation (429 atoms with edge length of 1.5 nm) was obtained from a larger one (1466 atoms with edge length of 2.3 nm), recently proposed by Liu and Di Valentin,[6] by simply reducing the total number of atoms. Both these models are enclosed by six (001) facets, as observed in experiments,[7,8] and present the same type of corners, edges, and surface reconstruction, which consists of the transfer of three six-coordinated iron atoms from octahedral to tetrahedral sites at four of the eight corners of the cube, i.e., those exposing tetrahedral Fe atoms. For more details regarding this reconstruction process, please refer to Ref. 6. The total magnetization is computed according to the formula, which determines the total magnetic moment of a magnetite system, as proven in Ref. 6:

$$m_{tot} = 5 \times \left[N\left(Fe^{3+}_{Oct}\right) - N(Fe^{3+}_{Tet})\right] + 4 \times \left[N\left(Fe^{2+}_{Oct}\right) - N(Fe^{2+}_{Tet})\right], \quad (1)$$

where Fe^{3+}_{Oct} and Fe^{2+}_{Oct} are Fe^{3+} and Fe^{2+} ions at octahedral sites, Fe^{3+}_{Tet} and Fe^{2+}_{Tet} are Fe^{3+} and Fe^{2+} ions at tetrahedral sites and N is the number of the corresponding ions. Similarly to what happens in bulk magnetite, for Fe^{3+}_{Oct} and Fe^{3+}_{Tet} the high-spin $3d^5$ configuration gives an atomic magnetic moment of +5 and -5 μ_B, respectively; for Fe^{2+}_{Oct} and Fe^{2+}_{Tet} the high-spin $3d^6$ electron configuration gives +4 and -4 μ_B, respectively. The total magnetic moment m_{tot} was found to be 288 μ_B for the cubic NP under investigation.

To mimic spin disorder phenomena, we forced some Fe^{3+}_{Oct} to hold a negative atomic magnetic moment of -5 μ_B, instead of +5 μ_B,[9,10] which lowers the total magnetic moment of the NP. We named the energy difference between the spin-up and spin-down solutions as $\Delta E_{Spin-Flip}$. After the spin-flip, we allowed for a full atomic relaxation of the NP, and we named the energetic gain associated to the relaxation as $\Delta E_{Relaxation}$. We named the sum of these two contributions as ΔE_{SF+Rel}:

$$\Delta E_{SF+Rel} = \Delta E_{Spin-Flip} + \Delta E_{Relaxation}. \quad (2)$$

For further details regarding the spin-flip process, please refer to Ref. 10 and corresponding Supplementary Material.

The adsorption energy per adsorbed molecule (E_{ads}) was calculated as follows:

$$E_{ads} = (E_{total} - E_{NP} - N_{molecule} \times E_{molecule})/N_{molecule}, \quad (3)$$

where E_{total} is the total energy of the whole system (NP and adsorbed molecules), E_{NP} is the energy of the Fe_3O_4 NP, $N_{molecule}$ is the number of adsorbed molecules and $E_{molecule}$ is the energy of one isolated molecule. This formula provides a value for the adsorption energy that is normalized by the total number of adsorbed molecules.

The band center of mass (COM) was computed using the formula:[11,12,13]

$$band\ COM = \frac{\int_{-\infty}^{E_F} E\rho(E)dE}{\int_{-\infty}^{E_F} \rho(E)dE}, \quad (4)$$

where E is the energy, $\mathrm{E_F}$ the Fermi energy (which is set to 0), and $\rho(\mathrm{E})$ the electronic density of states.

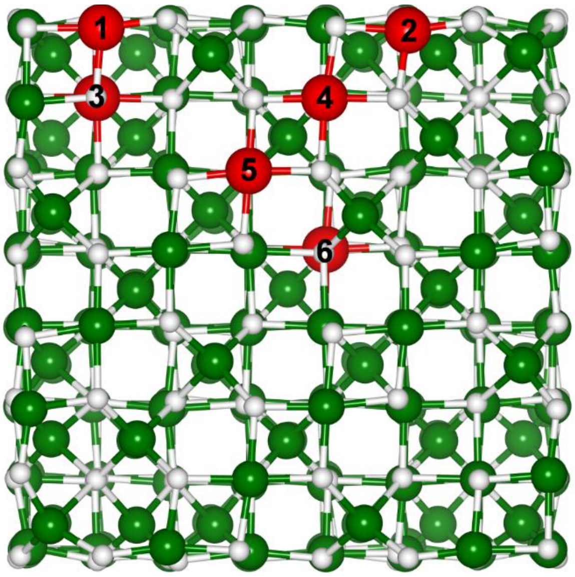

Figure S1. Global minimum energy structure of the magnetite cubic NP. The white, green, and labelled red beads represent O, Fe, and Fe_{Oct} on which the spin-flip cost is evaluated, respectively. In the nomenclature used for defining the spin-flipped Fe_{Oct} ions, further labels *4c-6c* indicate the actual coordination number of the corresponding ions.

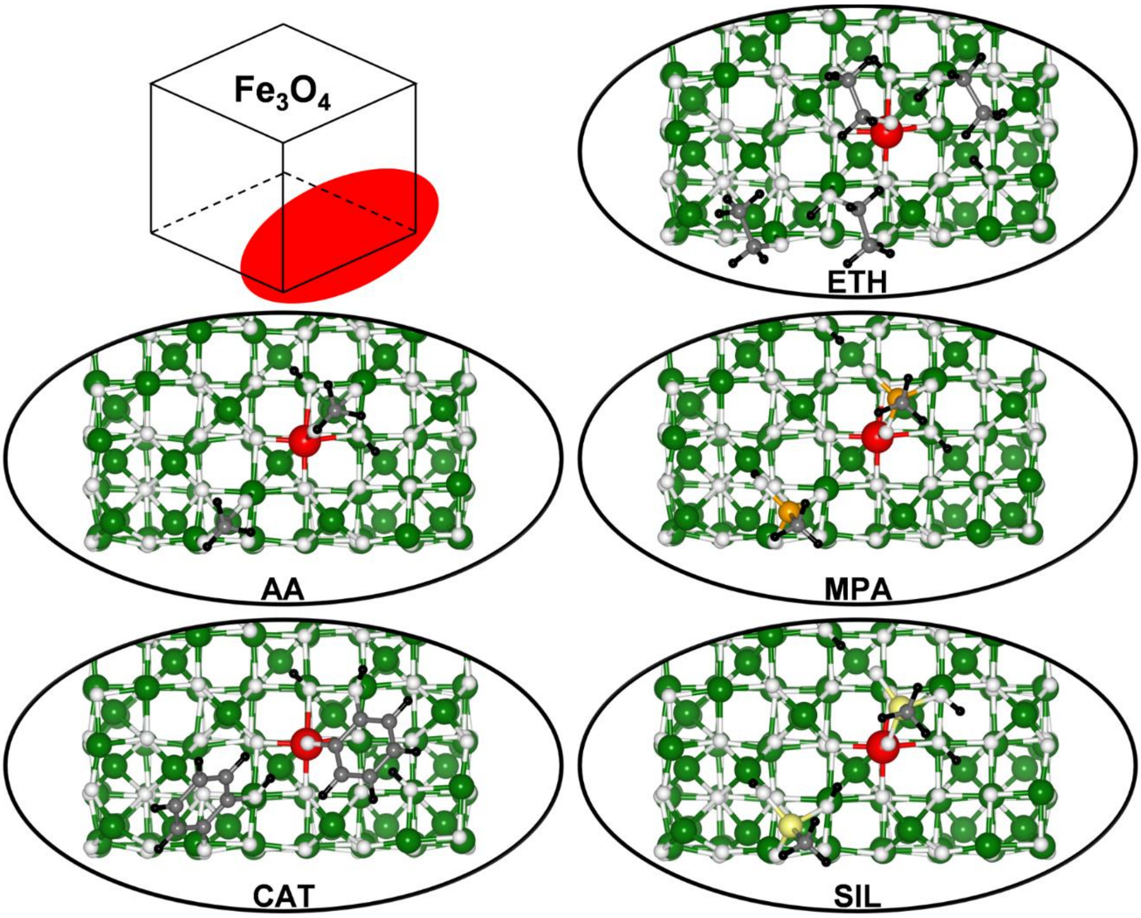

Figure S2. Ball-and-stick representations for the adsorption of different ligands at local high coverage onto the nanoparticle surface. Only the ligands and the NP surface portion around $Fe_{Oct}^{3+}5c-(\mathbf{5})$ are shown for clarity, as schematized in top left corner of the figure. The black, gray, orange, yellow, white, green, and red beads represent H, C, P, Si, O, Fe, and Fe_{Oct} on which the spin-flip process is investigated, respectively.

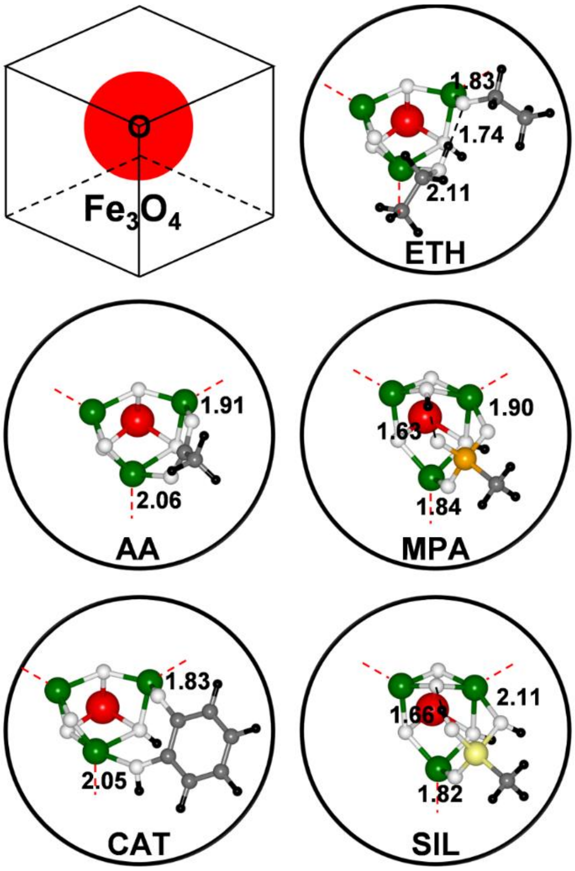

Figure S3. Ball-and-stick representations for the most stable adsorption mode of different ligands at local low coverage onto the NP corner. Only the ligands and the NP O-corner are shown for clarity, as schematized in top left corner of the figure. The red dashed lines indicate the edges of the hidden NP. The black dashed lines indicate the formation of hydrogen bonds. The H-bonds and Fe-O_{Ligand} bonds length (in Å) are shown. The black, gray, orange, yellow, white, green, and red beads represent H, C, P, Si, O, Fe, and Fe_{Oct} on which the spin-flip process is investigated, respectively.

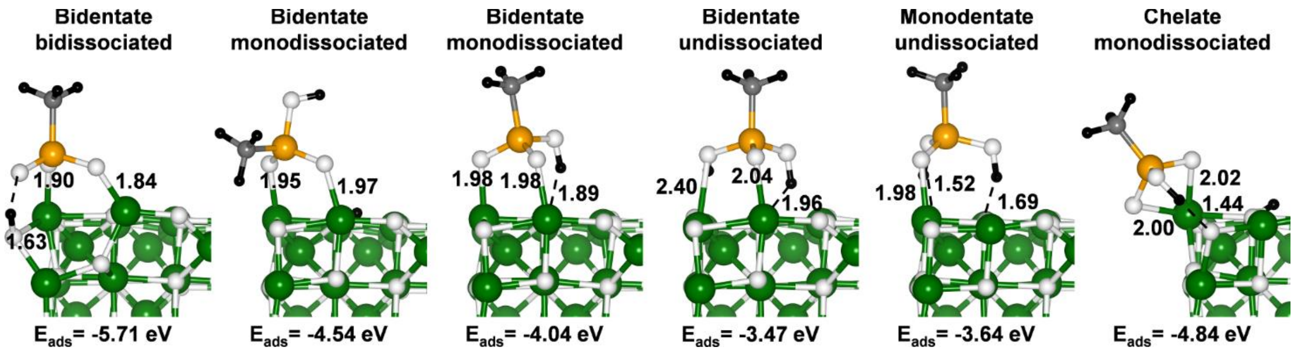

Figure S4. Ball and stick representations of the Fe_3O_4 cubic NP with a methylphosphonic acid (MPA) molecule adsorbed on its corner in different (stable) configurations. The black, gray, orange, white, and green beads represent H, C, P, O, and Fe, respectively. H-bonds are indicated by dashed black lines. The H-bonds and Fe-O_{MPA} bonds length (in Å) and the adsorption energy (in eV) calculated at HSE06 level are shown.

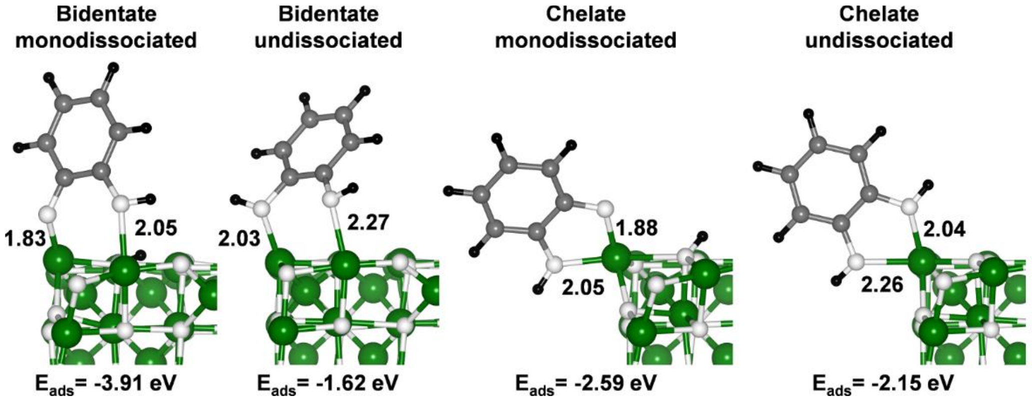

Figure S5. Ball and stick representations of the Fe_3O_4 cubic NP with a catechol (CAT) molecule adsorbed on its corner in different (stable) configurations. The black, gray, white, and green beads represent H, C, O, and Fe, respectively. The Fe-O_{CAT} bonds length (in Å) and the adsorption energy (in eV) calculated at HSE06 level are shown.

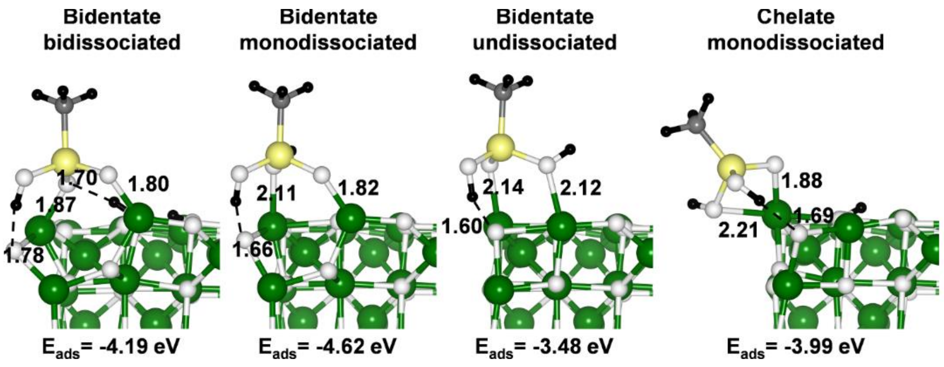

Figure S6. Ball and stick representations of the Fe_3O_4 cubic NP with a methylsilanetriol (SIL) molecule adsorbed on its corner in different (stable) configurations. The black, gray, yellow, white, and green beads represent H, C, Si, O, and Fe, respectively. H-bonds are indicated by dashed black lines. The H-bonds and Fe-O_{SIL} bonds length (in Å) and the adsorption energy (in eV) calculated at HSE06 level are shown.

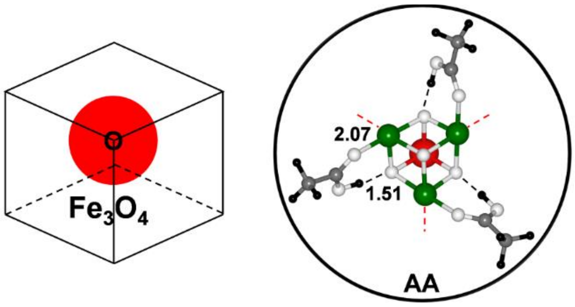

Figure S7. Ball-and-stick representation for the adsorption of AA in the monodentate mode at local high coverage onto the NP corner. Only the ligands and the NP O-corner are shown for clarity, as schematized on the left. The red dashed lines indicate the edges of the hidden NP. The black dashed lines indicate the formation of hydrogen bonds. The H-bond and the Fe-O_{Ligand} bond lengths (in Å) for the asymmetric unit are shown. The black, gray, white, green, and red beads represent H, C, O, Fe, and Fe_{Oct} on which the spin-flip process is investigated, respectively.

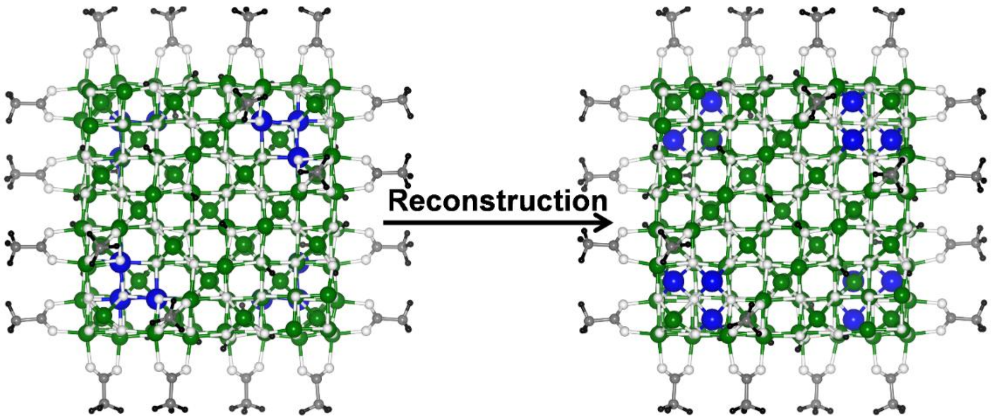

Figure S8. Ball-and-stick representations of the minimum energy structures of the unreconstructed (left) and reconstructed (right) NP at half acetic acid coverage (24 acetic acid molecules). The black, gray, white, green, and blue beads represent H, C, O, Fe, and Fe_{Oct} which are involved in the reconstruction process, respectively. The reconstructed NP is favored by about -1.2 eV.

Table S1. Spin-flip energetics of pairs of Fe ions in comparison with those for single Fe ions from Ref. 10.

	$\Delta E_{Spin\text{-}Flip}$ (eV)	$\Delta E_{Relaxation}$ (eV)	ΔE_{SF+Rel} (eV)
$Fe^{3+}_{Oct}-(\mathbf{1},\mathbf{3})$	**+358**	**-99**	**+260**
$Fe^{3+}_{Oct}4c-(\mathbf{1})$	+252	-77	+174
$Fe^{3+}_{Oct}6c-(\mathbf{3})$	+270	-256	+13
$Fe^{3+}_{Oct}-(\mathbf{4},\mathbf{5})$	**+607**	**-112**	**+495**
$Fe^{3+}_{Oct}5c-(\mathbf{4})$	+425	-88	+337
$Fe^{3+}_{Oct}5c-(\mathbf{5})$	+208	-71	+137

The energy cost for the simultaneous spin-flip of nearby Fe ions pair $Fe^{3+}_{Oct}4c-(\mathbf{1})$ / $Fe^{3+}_{Oct}6c-(\mathbf{3})$ is slightly higher (ca 70 meV) than the sum of those for the two independent single process, indicating that there is no cooperation. For the $Fe^{3+}_{Oct}5c-(\mathbf{4})$ / $Fe^{3+}_{Oct}5c-(\mathbf{5})$ pair, the cost for the simultaneous spin-flip corresponds almost exactly to the sum of those for the two independent single process (with a small difference of ca 20 meV). Based on these results, we conclude that the simultaneous spin-flips of multiple Fe centers, even when they are close, are not cooperative processes, in analogy to what observed for Fe ions in distant position in our previous work.[10]

References

[1] Krukau, A. V.; Vydrov, O. A.; Izmaylov, A. F.; Scuseria, G. E. Influence of the exchange screening parameter on the performance of screened hybrid functionals. *J. Chem. Phys.* **2006**, *125*(22), 224106.

[2] Dovesi, R.; Erba, A.; Orlando, R.; Zicovich-Wilson, C. M.; Civalleri, B.; Maschio, L.; Rerat, M.; Casassa, S.; Baima, J.; Salustro, S.; Kirtman, B. Quantum-mechanical condensed matter simulations with CRYSTAL. *Wiley Interdiscip. Rev. Comput. Mol. Sci.* **2018**, *8*(4), e1360.

[3] Dovesi, R.; Saunders, V. R.; Roetti, C.; Orlando, R.; Zicovich-Wilson, C. M.; Pascale, F.; Civalleri, B.; Doll, K.; Harrison, N. M.; Bush, I. J.; D'Arco, P.; Llunell, M.; Causà, M.; Noël, Y.; Maschio, L.; Erba, A.; Rerat, M.; Casassa, S. CRYSTAL17 User's Manual, University of Torino, Torino, 2017.

[4] Liu, H.; Di Valentin, C. Band gap in magnetite above Verwey temperature induced by symmetry breaking. *J. Phys. Chem. C* **2017**, *121*(46), 25736-25742.

[5] Liu, H.; Di Valentin, C. Bulk-terminated or reconstructed Fe_3O_4(001) surface: Water makes a difference. *Nanoscale* **2018**, *10*(23), 11021-11027.

[6] Liu, H.; Di Valentin, C. Shaping magnetite nanoparticles from first principles. *Phys. Rev. Lett.* **2019**, *123*(18), 186101.

[7] Kovalenko, M. V.; Bodnarchuk, M. I.; Lechner, R. T.; Hesser, G.; Schäffler, F.; Heiss, W. Fatty acid salts as stabilizers in size-and shape-controlled nanocrystal synthesis: the case of inverse spinel iron oxide. *J. Am. Chem. Soc.* **2007**, *129*(20), 6352-6353.

[8] Kim, D.; Lee, N.; Park, M.; Kim, B. H.; An, K.; Hyeon, T. Synthesis of uniform ferrimagnetic magnetite nanocubes. *J. Am. Chem. Soc.* **2009**, *131*(2), 454-455.

[9] Herng, T. S.; Xiao, W.; Poh, S. M.; He, F.; Sutarto, R.; Zhu, X.; Li, R.; Yin, X.; Diao, C.; Yang, Y.; Huang, X.; Yu, X.; Feng, Y. P.; Rusydi, A.; Ding, J. Achieving a high magnetization in sub-nanostructured magnetite films by spin-flipping of tetrahedral Fe^{3+} cations. *Nano Res.* **2015**, *8*(9), 2935-2945.

[10] Bianchetti, E.; Di Valentin, C. Mechanism of spin ordering in Fe_3O_4 nanoparticles by surface coating with organic acids. *Mater. Today Nano* **2022**, *17*, 100169.

[11] Gajdoš, M.; Eichler, A.; Hafner, J. CO adsorption on close-packed transition and noble metal surfaces: trends from ab initio calculations. *J. Phys. Condens. Matter* **2004**, *16*(8), 1141.

[12] Stroppa, A.; Kresse, G. The shortcomings of semi-local and hybrid functionals: what we can learn from surface science studies. *New J. Phys.* **2008**, *10*(6), 063020.

[13] Hulva, J.; Meier, M.; Bliem, R.; Jakub, Z.; Kraushofer, F.; Schmid, M.; Diebold, U.; Franchini, C.; Parkinson, G. S. Unraveling CO adsorption on model single-atom catalysts. *Science* **2021**, *371*(6527), 375-379.
